# Customized treatment protocols for patients with closed fracture in hospitals at varying coronavirus disease 2019 (COVID-19) risk

**DOI:** 10.1186/s12891-021-04614-w

**Published:** 2021-08-30

**Authors:** Yu He, Zhong-Hua Guo, Yong-Guang Wu, Rui Li, Xie Xie, De-Hao Fu

**Affiliations:** 1 Department of Orthopedics, Union Hospital, Tongji Medical College, Huazhong University of Science and Technology (HUST), 1277 Jiefang Avenue, 430022 Wuhan, P.R. China; 2 Department of Orthopedics, People’s Hospital of Dongxihu District, Wuhan, P.R. China; 3 Department of Orthopedics, People’s Hospital of Caidian District, Wuhan, P.R. China; 4 Department of Traditional Chinese Medicine, Wuhan Third Hospital, Tongren Hospital of Wuhan University, 430060 Wuhan, P.R. China

**Keywords:** Coronavirus disease 2019 (COVID-19), Risk level of the epidemic, Delayed surgery, Closed fracture, Treatment protocol, Optimization strategy

## Abstract

**Background:**

To determine an optimized treatment protocol during the COVID-19 epidemic for patients with closed fracture and delayed surgery.

**Methods:**

The epidemic data of three hospitals, randomly selected from different administrative regions of Wuhan, were analyzed retrospectively from 23 January to 31 March 2020. Changes in the number of confirmed cases per day (cumulative and new) of each region were tracked as a reflection of changing epidemic risk levels. The risk level map was drawn. The epidemic status, treatment protocols, and treatment efficiencies for patients with closed fracture in the three hospitals were compared.

**Results:**

Overall, 138 patients with closed fracture were admitted. Each hospital had established its own protocol, according to the initial perceived risk. Based on the risk level map, over the study period, the risk levels of the three regions changed independently and were not in sync. All patients recovered and were timely discharged. No staff member was detected with COVID-19.

**Conclusions:**

The COVID-19 risk level of each area is dynamic. To optimize medical resources, avoid cross-infection, and improve efficiency, changes in epidemic risk should be monitored. For patients with closed fracture, treatment protocols should be adjusted according to changes in epidemic risk.

## Background

The novel coronavirus pneumonia due to SARS-CoV-2 (severe acute respiratory syndrome coronavirus 2) infection was termed coronavirus disease 2019 (COVID-19) by the World Health Organization (WHO) [1]. To prevent the spread of the disease, the local government of Wuhan took decisive action to shut down the city. The imposed limits on travel let to a sharp reduction in traffic injuries. Even so, low-energy fractures (i.e., due to falling from up to standing height [2]) still occurred from time to time during the home quarantine period.

Insufficient protection in the midst of an epidemic can leave medical staff vulnerable to infection. For example, a postoperative patient with delayed diagnosis of COVID-19 caused the transmission of the disease to 14 of the associated medical personnel in one ward [3]. In 2003 in Canada during the SARS (severe acute respiratory syndrome) epidemic, nine medical staff were infected after exposure to an infected patient in the operating room [4]. During the COVID-19 pandemic, these unfortunate experiences led to a global guideline for limiting elective surgical procedures, in order to reduce the risk of cross-infection, and make medical space and personnel more available for the greater need [5].

However, patients with severe fracture, such as displacement or intraarticular, have absolute indications for surgery, and conservative treatment cannot achieve satisfactory results [6]. Although it is unclear if SARS-CoV-2 can harbor in synovial fluid, musculoskeletal symptoms such as myalgia, arthralgia, and asthenia are very common. To relieve musculoskeletal symptoms, joint (intra-articular and periarticular) injection therapy with hyaluronic acid can be used [7]. Yet, it can be difficult to diagnosis COVID-19 immediately in the emergency room because the disease has a long incubation period, mild symptoms go unrecognized, and nucleic acid detection is prone to a false negative result. Therefore, under epidemic conditions, it is challenging to provide timely, safe, and effective treatment to patients in need of trauma orthopedic surgery.

The objective of the present study was to compare the treatment protocols at three hospitals with various levels of risk and to determine an optimized strategy of treatment during the COVID-19 epidemic for patients with closed fracture and delayed surgery.

## Methods

This is a retrospective cohort study. The clinical research ethics board of Union Hospital, Tongji Medical College, and Huazhong University of Science and Technology approved the protocol of the study (No. LSZ [174], 2020) and all methods were performed in accordance with the relevant guidelines and regulations. This report conforms to the recommendations of STROBE (Strengthening the Reporting of Observational Studies in Epidemiology) [8].

### Setting and participants

The study was performed based on data collected, for the period from 23 to 2020 (the beginning of Wuhan lockdown) to 31 March 2020, from the following three hospitals: Wuhan Union Hospital (H1); People’s Hospital of Dong-Xi-Hu District, Wuhan (H2); and People’s Hospital of Caidian District, Wuhan (H3). These hospitals are located in different administrative regions of Wuhan and were randomly selected according to their probable risk levels, i.e., H1 is located in the Jianghan District, which was the most affected area of the epidemic in Wuhan. H2 is located at the edge of the third ring of Wuhan, and H3 is in the far urban area of Wuhan.

We included patients with closed fracture in delayed surgery, without restriction for age or gender. Patients who required emergency treatment due to the following were excluded from this analysis: open fracture, or a few closed fractures or fracture-dislocation including those with neurovascular injury or osteofascial compartment syndrome, or spine fractures with spinal cord injury. In addition, patients with confirmed COVID-19 were excluded.

### Data collection

The official data for the cumulative number of confirmed cases and newly confirmed cases in each administrative district of Wuhan were obtained from the Wuhan Municipal Health Commission. The primary clinical characteristics of the patients at the three hospitals were collected, as well as the treatment protocols (screening, anesthesia, and operation); and preoperative, postoperative, and total hospitalization times. The patients were treated as three cohorts (P1, P2, P3), corresponding to the three respective hospitals (H1, H2, H3).

### Data analysis

The risk category of each county or urban unit was judged as low, moderate, or high. Low risk was defined as no confirmed cases, or no newly confirmed cases for 14 consecutive days. Moderate risk consisted of ≤ 50 cumulative newly confirmed cases within 14 days, or ≤ 50 cumulative confirmed cases without a clustering epidemic within 14 days. High risk meant that the cumulative number of confirmed cases was > 50 with a clustering epidemic, within 14 days.

**Fig. 1:**
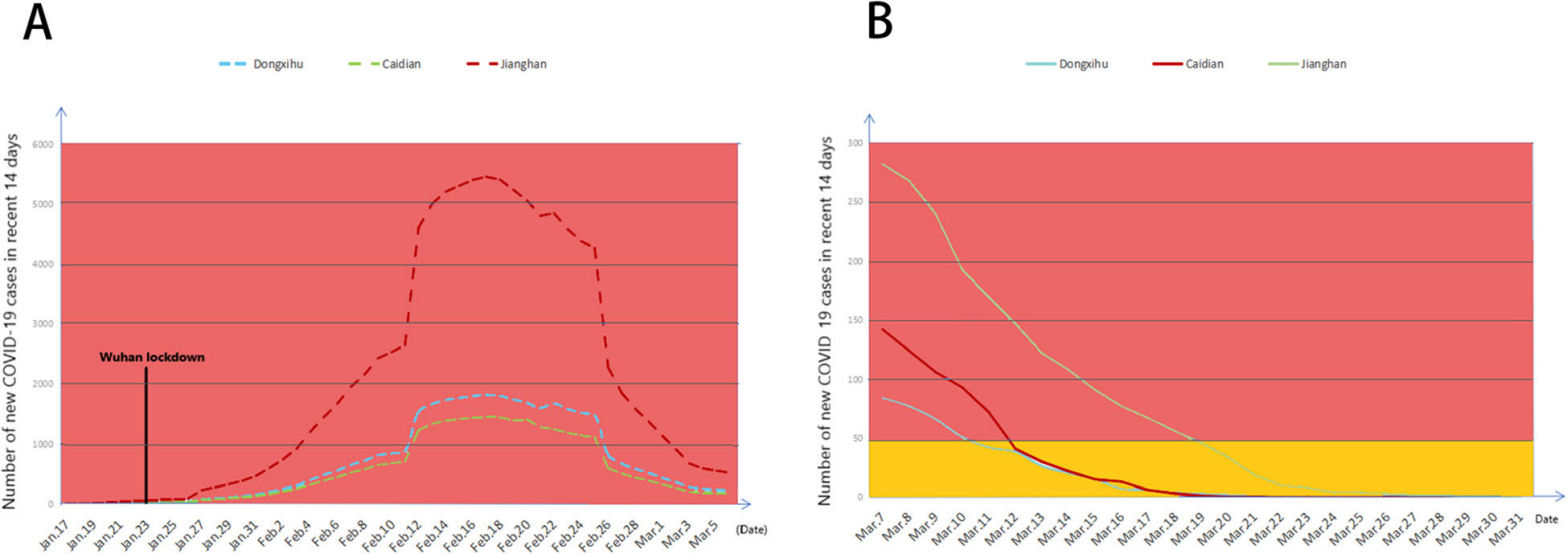
Dynamic changes in the time axis of the epidemic risk in the three regions (based on the cumulative number of newly diagnosed people within 14 days). (**A**) From January 17, 2020 to March 5, 2020. (**B**) From March 7, 2020 to March 31, 2020

The dynamic chart of the COVID-19 epidemic risk level was recorded based on the government official notification data. The percentage of new confirmed cases per million population in each administrative region was calculated daily, and the newly confirmed number was added every day. A risk level map of the COVID-19 data in Wuhan was drawn with the location of the three hospitals (H1, H2, H3) to assess the daily risk level of COVID-19 in each administrative district. The risk level for COVID-19 in different regions was compared on the time axis (Fig. 1).

Flow charts for each treatment protocol in the three hospitals were drafted. Categorical variables are shown as count, with percentage or frequency. Continuous variables are shown as mean with standard deviation. For comparisons of categorical variables, Pearson’s chi-squared test or Fischer’s test were used. Comparisons of continuous variables among the three hospitals were performed by analysis of variance (ANOVA) or Kruskal-Wallis one-way analysis-of-variance, depending on the distribution of the data. All statistical analyses were performed using SPSS 20.0 statistical software.

## Results

### Clinical characteristics of the included patients

Overall, 138 patients with closed fracture were admitted to the three hospitals during the study period (Table 1). The age of these patients was 52.4 ± 16.6 years, and 68 of them were men (49.3 %). The predominant three fractures were of the hip, tibiofibular joint, and femur (28.6 %, 21.1 %, and 11.3 %, respectively, or 38, 28, and 15 patients).

**Table. 1:** Clinical characteristics of the 133 patients with closed fracture ^a^

	H1	H2	H3	Total	P ^b^
Subjects, n	32	45	61	138	—
Age, y	46.1 ± 19.4	57.1 ± 17.6	53.0 ± 13.7	52.4 ± 16.6	0.001
Male, n (%)	18 (56.3)	21 (46.7)	29 (47.5)	68 (49.3)	0.656
Fracture					
Total, n	35	56	68	159	
Tibia and fibula	3 (8.6)	9 (16.1)	16 (23.5)	28 (17.6)	0.207
Femoral neck	5 (14.3)	11 (19.6)	7 (10.3)	23 (14.5)	0.032
Femur	5 (14.3)	5 (8.9)	5 (7.4)	15 (9.4)	0.393
Spinal column	8 (22.9)	4 (7.1)	1 (1.5)	13 (8.1)	0.023
Ankle	1 (2.9)	6 (10.7)	6 (8.8)	13 (8.1)	0.238
Calcaneus	2 (5.7)	2 (3.6)	7 (10.3)	11 (6.9)	0.035
Patella	1 (2.9)	4 (7.1)	4 (5.9)	9 (5.7)	0.484
Clavicle	2 (5.7)	1 (1.8)	6 (8.8)	9 (5.7)	0.163
Humerus	1 (2.9)	2 (3.6)	5 (7.4)	8 (5.0)	0.047
Intertrochanteric femur	0	5 (8.9)	3 (4.4)	8 (5.0)	0.069
Pelvis & acetabulum	4 (11.4)	2 (3.6)	1 (1.5)	7 (4.4)	0.159
Ulna and radius	1 (2.9)	2 (3.6)	4 (5.9)	7 (4.4)	0.301
Other	2 (5.7)	3 (5.4)	3 (4.4)	8 (5.0)	0.742
Anesthesia					
Total, n	30	42	61	133	
General	25 (83.3)	5 (11.9)	5 (8.2)	35 (26.3)	0.003
Brachial plexus	1 (13.3)	6 (14.3)	14 (23.0)	21 (15.8)	0.237
Spinal & epidural	4 (13.3)	31 (73.8)	42 (68.9)	77 (57.9)	0.048
Operation type					
Total, n	32	42	61	135	
Plate and screw	26 (81.3)	33 (78.6)	50 (82.0)	109 (80.7)	0.002
External fixator	2 (6.3)	0	2 (3.3)	4 (3.0)	0.718
Joint replacement	4 (12.5)	5 (11.9)	5 (8.2)	14 (10.4)	0.029
Intramedullary nail	0	4 (9.5)	4 (6.6)	8 (5.9)	0.001
Hospital stay, d					
Total	21.9 ± 8.1	19.5 ± 4.5	15.3 ± 4.3	18.6 ± 5.8	
Preoperative	10.9 ± 6.1	8.9 ± 4.4	6.8 ± 4.1	8.5 ± 5.0	0.015
Postoperative	10.7 ± 3.9	6.8 ± 3.4	8.6 ± 3.2	8.9 ± 3.5	0.101

^a^ Reported as n (%) unless indicated otherwise.

^b^ P value comparing 3 target hospitals by ANOVA for continuous variables and chi-squared test for categorical variables.

Abbreviations: *H1* Wuhan Union Hospital; *H2* People’s Hospital of Dong-Xi-Hu District; *H3* People’s Hospital of Caidian District.

Most of the 138 patients were hospitalized for more than two weeks. During the 2-week isolation period, two patients and one caregiver were confirmed to have COVID-19 in H1, and three patients were confirmed in H2. These five patients and one caregiver were transferred to a designated hospital for COVID-19, for further treatment immediately. No medical staff member was found to have COVID-19 during the study period in any of the three hospitals.

### Treatment protocols in the three hospitals

#### At H1

**Fig. 2:**
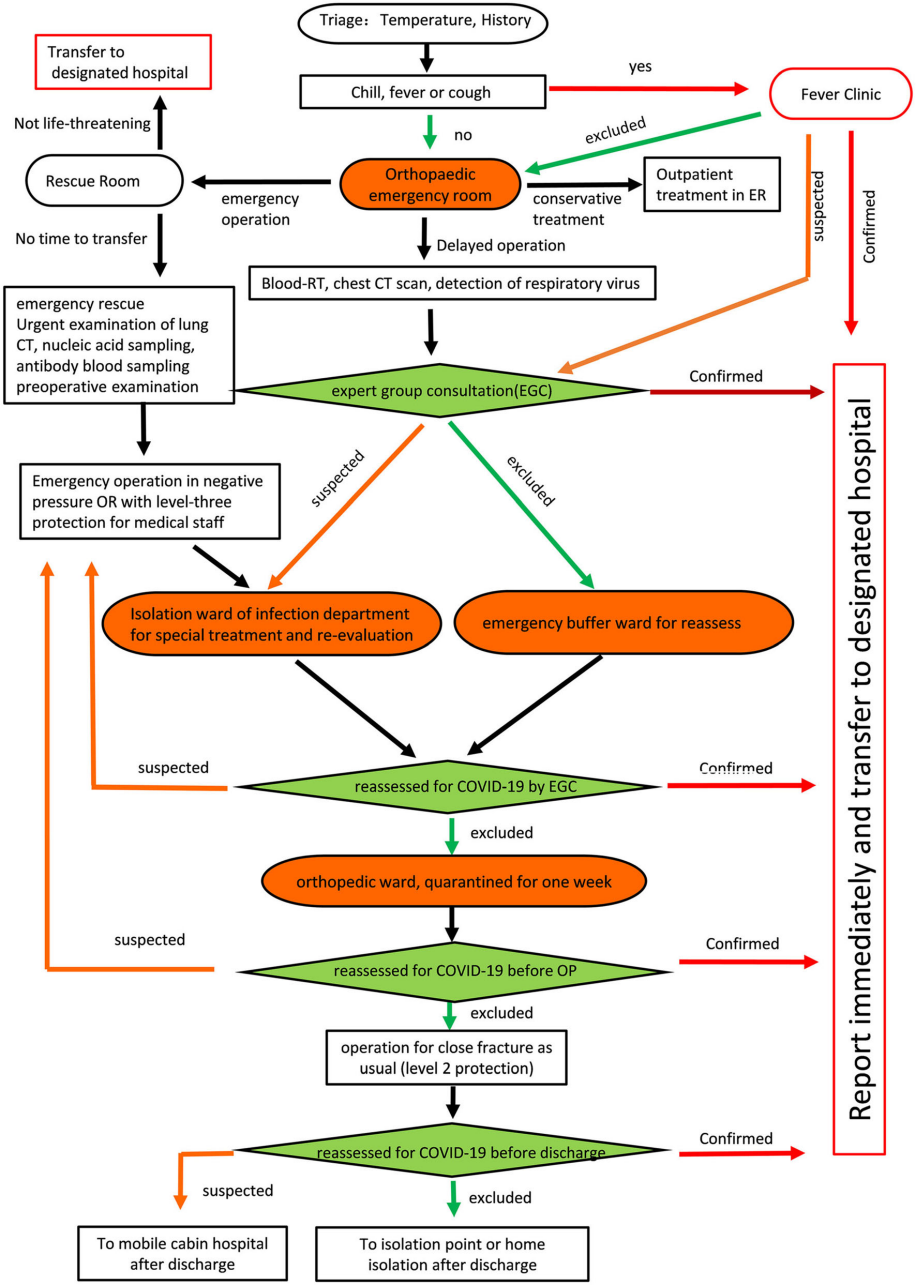
Flow chart of admission classification and treatment process for emergency patients in Wuhan Union Hospital (H1)

H1 is in the area of highest risk for COVID-19. To manage the epidemic, H1 established a COVID-19 expert consultation group and buffer wards. Especially, an orthopedic emergency room (OER) was set up to screen, separate, and treat patients with COVID-19 (Fig. 2).

The following protocol (P1) was applied at H1. First, outpatients were sorted at an emergency triage station. Those with fever or respiratory symptoms were guided to the fever clinic, and those with confirmed COVID-19 were transferred to a designated hospital. Second, all fracture cases without upper respiratory symptoms were treated in the OER, where preoperative preparation was performed for those who needed delayed surgery; mild cases were treated conservatively. Those patients with non-life-threatening conditions were transferred to a designated hospital. For patients at critical risk, emergency surgeries were performed in a negative pressure operating room under level-3 protection. After the operation, the patients were admitted to the isolation ward of the infection department.

Patients with fractures who required hospitalization for a selective operation were admitted to the buffer ward, after screening with chest computed tomography (CT) and for SARS-CoV-2 antibody. After 24 h, screening for SARS-CoV-2 was repeated in the buffer ward before patients were transferred to the regular orthopedic ward. After one week of isolation, patients in the orthopedic ward were reevaluated for the SARS-CoV-2 antibody. For those who tested negative, surgery was performed under routine protocols. Patients who tested positive were transferred to a designated hospital. Finally, all patients were reevaluated for the SARS-CoV-2 antibody before discharge.

#### At H2

Relative to H1, the epidemic risk level at H2 was considered lower because of its geographic location. The administration at H2 established an expert consultation group, but not a designated emergency orthopedic consulting room, and the emergency room served for patients’ reception.

**Fig. 3:**
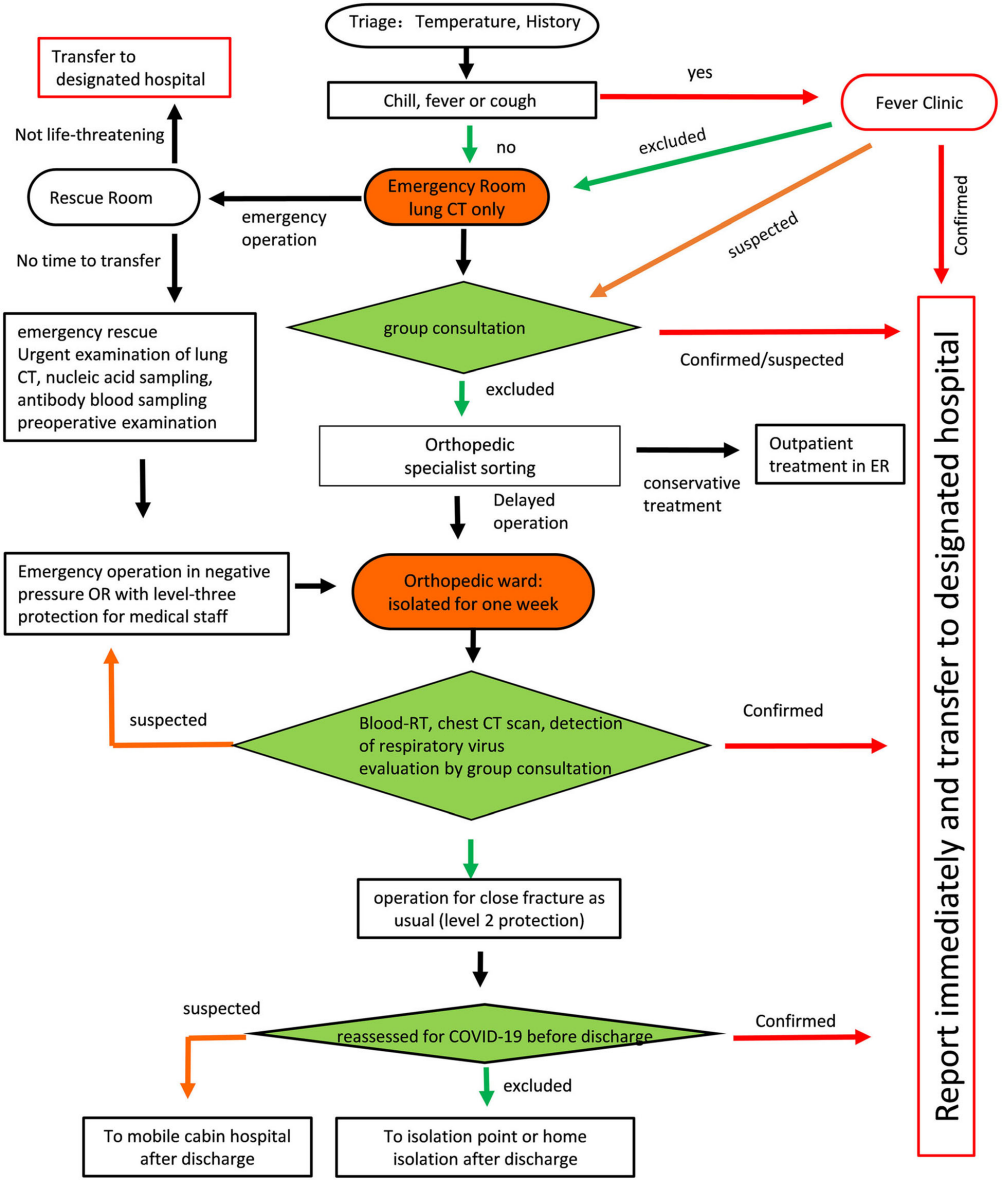
Flow chart of admission classification and treatment process for emergency patients in Dongxihu District Hospital (H2)

The patient protocol (P2) applied in H2 was as follows (Fig. 3). First, screening with emergency chest CT was performed for each patient in the emergency room, without detection of the virus nucleic acid or antibody. For patients whose CT was negative, the orthopedic clinic then addressed the problem of closed fracture and other orthopedic emergencies. There was no special buffer isolation ward set up at H2. Patients and their caregivers were isolated in a single room in the general ward.

After more than one week of admission isolation, patients were reexamined for COVID-19 by testing for virus nucleic acid and antibody. For patients were negative results, surgery was performed. Expert diagnosis and treatment of COVID-19 was emphasized. A team of experts was repeatedly invited for identification consultation, and relied on to ensure the accuracy of diagnosis and treatment.

#### At H3

**Fig. 4:**
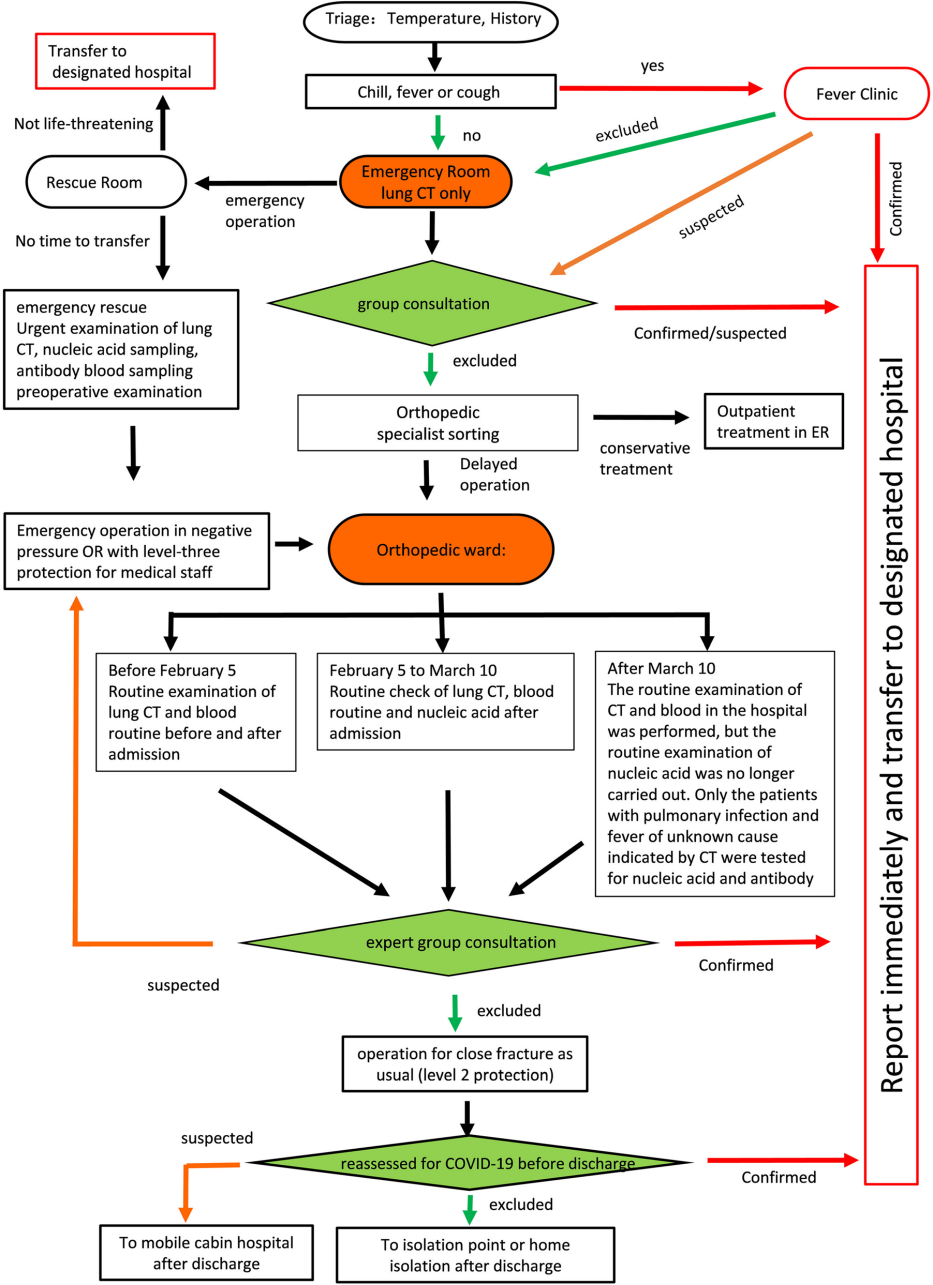
Flow chart of admission classification and treatment process for emergency patients in Caidian District Hospital (H3)

Because H3 is located in the remote urban area of Wuhan, the epidemic risk was assumed lower than at H1 or H2. A significant feature of the protocol (P3) applied at H3 was that all treatments were adjusted based on the dynamic changes of its own test conditions and the epidemic at the hospital location (Fig. 4).

Prior to 5 February 2020, it was impossible to perform a routine nucleic acid test at H3, and admission screening did not include nucleic acid screening. (Since 5 February 2020, the hospital has been able to check for the nucleic acid.) Surgical patients were screened by chest CT, routine blood, and nucleic acid test (twice) within three days before surgery. If the result was negative, patients underwent standard operation protocols; if positive, patients were transferred to the designated hospital. Since no patient was found with a positive nucleic acid test from 5 February to 10 March, H3 has not routinely checked for nucleic acid since 10 March 2020. To shorten the diagnostic and treatment protocols, and improve efficiency, H3 only performed the nucleic acid test and antibody test for patients with pulmonary infection and fever of unknown cause.

### Anesthesia, operation, and prognosis

The anesthesia type that was most frequently applied at H1 was general anesthesia (83.3 %; Table 1). However, at H2 and H3, combined spinal and epidural anesthesia was preferred, at rates of 73.8 and 68.9 %, respectively. All fractures were treated with classic surgical methods. Most fractures were treated with open reduction and internal fixation with a steel plate, some with closed reduction and internal fixation with an intramedullary nail. A few were treated with an external fixator or artificial joint replacement.

The hospital stay at H1 was significantly longer than at H2 or H3 (*P* < 0.05), including the length of preoperative and the postoperative stay (Table 1). After surgery, all patients experienced a good alignment, and their wounds were well recovered. There were no perioperative complications, such as infection.

### Risk level map of COVID-19 in Wuhan

**Fig. 5:**
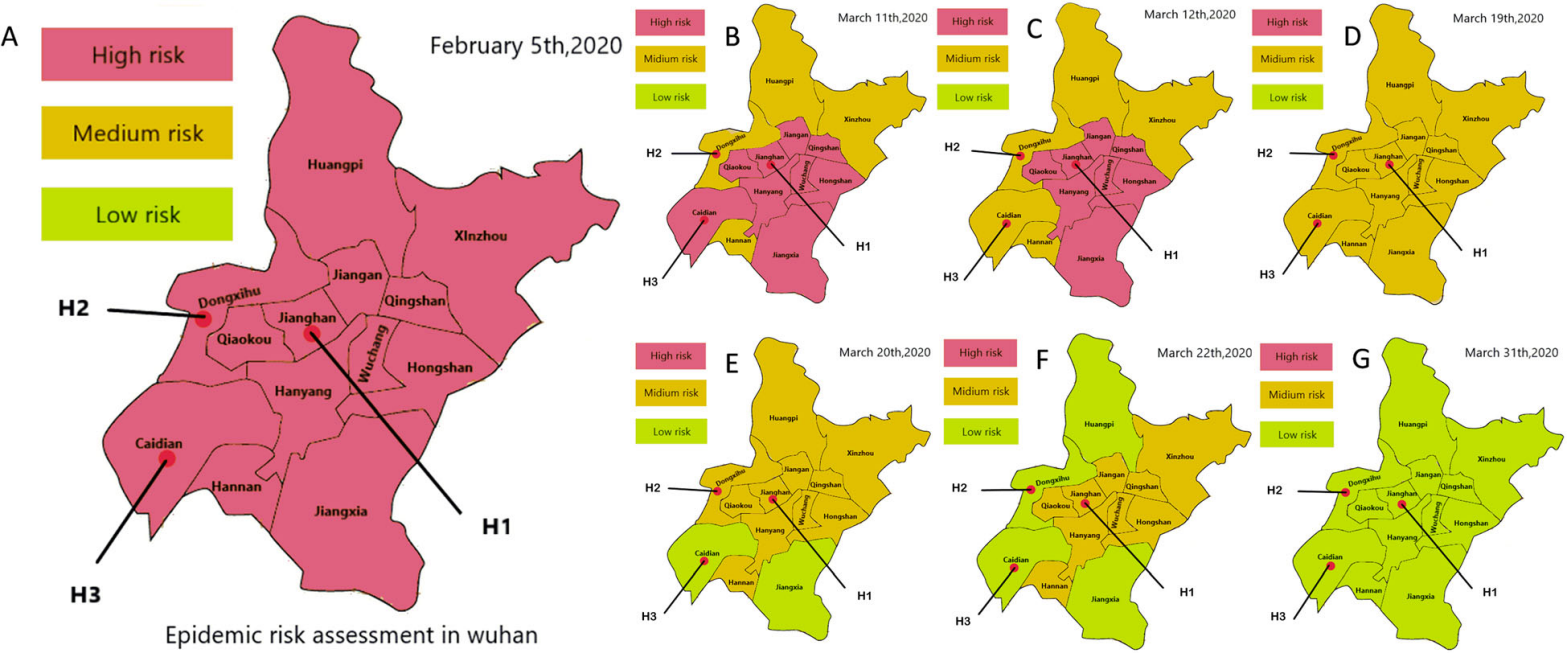
Risk level map of COVID-19 in Wuhan on some dates during pandemic. Wuhan Union Hospital (H1), Dongxihu District Hospital (H2), and Caidian District Hospital (H3) are in the Jianghan, Dongxihu, and Caidian areas, respectively

H1, H2, and H3 are in the Jianghan, Dong-Xi-Hu, and Caidian areas, respectively (Fig. 5). From 5 February to 31 March 2020, the risk level of COVID-19 in all these administration areas decreased. Most of the time, the hierarchy of risk, from high to low, was Jianghan, Dong-Xi-Hu, and Caidian (Fig. 1). The number of newly confirmed cases within the most recent 14 days in the three areas decreased from 1 March to 3 March 2020. The peak time for new cases within the most recent 14 days was from 12 to 26, February 2020.

## Discussion

Unlike in other countries [9, 10], in China there was no national protocol for closed fracture treatment during the pandemic. Multiple trauma care is often challenging too [11]. The present study explored a reasonable treatment protocol for patients with closed fractures during the COVID-19 pandemic by evaluating the results from 3 hospitals in Wuhan, China. The three hospitals adjusted their treatment protocol according to the level of epidemic risk and their own infrastructure. We found that it is feasible and effective to evaluate the epidemic risk level of each county and city, to select the appropriate protocols according to the risk level of each hospital, and to adjust protocols as changes in risk level occur. The above adjustment is consistent with the global guidance for surgical care during the COVID-19 pandemic [5].

One of our principles is that COVID-19 should not be a contraindication for surgery in patients with fracture [12]. According to our maps of dynamic COVID-19 risk levels, the three targeted hospitals differed in risk and source of patients. Patients at H2 and H3 are mainly local residents, while H1 is a large-scale tertiary hospital with patients from all areas of Hubei Province and with relatively complex cases. As the most severe area of the epidemic, H1 was overwhelmed during the entire period. During the pandemic, patients in non-designated hospitals with fracture who needed emergency surgery were simply bandaged and stabilized, and then transferred to designated hospitals for treatment [5, 13]. Concerning the cohort at H1, all patients who underwent immediate emergency surgery were treated as though they had confirmed COVID-19.

It is believed that close contact with patients’ infected respiratory droplets are the main routes of COVID-19 transmission [14], even when the infected person is asymptomatic [15, 16]. Some patients have no respiratory symptoms but do have diarrhea, mild muscle ache, or other symptoms, and already have the ability to transmit COVID-19 [17, 18]. In patients with acute fracture symptoms such as trauma, bleeding, or pain, asymptomatic COVID-19 can be easily overlooked [19, 20]. Musculoskeletal symptoms are also associated with COVID-19 [21]. Therefore, it is vital to enhance detection of COVID-19 and temporarily isolate patients after hospital admission.

According to the management guideline for fractures [19], waiting time for soft tissue swelling that is generally 1 to 2 weeks [22]. Notably, the incubation period of COVID-19 is also usually up to 2 weeks [23]. Therefore, we can make full use of the waiting period in patients with fracture to detect COVID-19. In our study, the overall wait time in each hospital was more than one week. Five patients (2 in H1, and 3 in H2) were found to have COVID-19 during the hospital stay prior to surgery.

Tracheal intubation to implement general anesthesia requires opening the patient’s respiratory tract, which increases the exposure risk of medical staff to the virus [24, 25]. During the pandemic, it has been suggested that simple anesthesia methods should be chosen if possible, i.e., local or nerve block, rather than general anesthesia for any operation [26]. In the present study, H1 applied general anesthesia significantly more often than H2 or H3 did for closed fracture surgeries, because of differences in conventional anesthesia tradition. However, none of the staff in any hospital contracted COVID-19 due to operative exposure. Therefore, if the prevention and control measures are appropriate, all kinds of anesthesia are safe [26].

During the pandemic, most hospitals in China strictly adhered to the same protocols, which did not allow patients with fracture a timely operation. As these were applied without regard for patients’ specific circumstances, there was a serious decline in work efficiency and great waste occurred. A recently published review [27] suggested conservative treatment, as far as possible, for non-obligatory fracture and in lesser equipped centers. The present study supplies a guideline for the admission of patients with fracture that depends on the risk of COVID-19. The cohort at H3 had the shortest hospital stay, where the risk of COVID-19 was least. Thus, the protocol at H3 is safe for COVID-19 areas at low risk. The protocol at H1, where the risk of COVID-19 was consistently high, is relatively cumbersome and hospitalization time is prolonged, but it is the most logically safe and suitable for those circumstances.

Currently, the gradual availability of COVID-19 vaccines has greatly relieved the burden of the hospitals. However, the protocols in the present study are very suitable for future pandemic conditions. The limitations of the present study include small sample size and a brief study period. Future large-scale research is needed to confirm our findings.

## Conclusions

During the COVID-19 pandemic, the methods for assessing risk at the regional level should be based on the local number of confirmed patients. For patients with closed fracture, the protocols described herein are practical and feasible, as they were customized according to the epidemic risk level in the geographic area. For low-risk areas, a relatively aggressive and simplified protocol (P3) was adopted; for medium and high-risk areas, the protocols were rather more conservative (P1/P2). Risk levels can change, and treatment protocols should be adjusted accordingly to save human, material, and financial resources, as well as ensure medical safety.

## Data Availability

The datasets used and/or analysed during the current study are available from the corresponding author on reasonable request.

## Abbreviations

COVID-19: Coronavirus disease 2019
CT: Computed tomography
H1: Wuhan Union Hospital
H2: People’s Hospital of Dong-Xi-Hu District, Wuhan
H3: People’s Hospital of Caidian District, Wuhan
OER: Orthopedic emergency room
WHO: World Health Organization

## References

[1] Sun P, Lu X, Xu C, Sun W, Pan B. Understanding of COVID-19 based on current evidence. J Med Virol. 2020;92(6):548–51. 10.1002/jmv.25722. 10.1002/jmv.25722 PMC7228250 32096567

[2] Furtado S, Rodrigues A, Dias S, Branco JC, Canhão H. Self-reported low-energy fractures and associated risk factors in people with diabetes: A national population-based study. Diabetes Res Clin Pract. 2019;147:93–101. 10.1016/j.diabres.2018.11.015. 10.1016/j.diabres.2018.11.015 30481576

[3] Wang X, Jiang X, Huang Q, Wang H, Gurarie D, Ndeffo-Mbah M, et al. Risk factors of SARS-CoV-2 infection in healthcare workers: a retrospective study of a nosocomial outbreak. Sleep Med X. 2020;2:100028. 10.1016/j.sleepx.2020.100028. 10.1016/j.sleepx.2020.100028 PMC7554494 33860224

[4] Ofner M, Lem M, Sarwal S, Vearncombe M, Simor A. Cluster of severe acute respiratory syndrome cases among protected health care workers-Toronto, April 2003. Can Commun Dis Rep. 2003;29(11):93–7. 12794968

[5] COVIDSurg Collaborative. Global guidance for surgical care during the COVID-19 pandemic. Br J Surg. 2020;107(9):1097–103. 10.1002/bjs.11646. 10.1002/bjs.11646 PMC7262310 32293715

[6] Lu J, Ren Z, Liu X, Xu YJ, Liu Q. Osteoporotic Fracture Guidelines and Medical Education Related to the Clinical Practices: A Nationwide Survey in China. Orthop Surg. 2019;11(4):569–77. 10.1111/os.12476. 10.1111/os.12476 PMC6712375 31322836

[7] Oliva F, Vittadini F, Frizziero A, Costantino C, Fusco A, Giai Via A (2020). IS Mu. LT Reccomendations for Intra and Periarticular Injections during COVID19 Pandemic. Muscles, Ligaments and. Tendons Journal.

[8] von Elm E, Altman DG, Egger M, Pocock SJ, Gøtzsche PC, Vandenbroucke JP (2007). The Strengthening the Reporting of Observational Studies in Epidemiology (STROBE) statement: guidelines for reporting observational studies. PLoS medicine.

[9] Luceri F, Morelli I, Accetta R, Mangiavini L, Maffulli N, Peretti GM (2020). Italy and COVID-19: the changing patient flow in an orthopedic trauma center emergency department. . J Orthop Surg Res.

[10] DiFazio LT, Curran T, Bilaniuk JW, Adams JM, Durling-Grover R, Kong K (2020). The Impact of the COVID-19 Pandemic on Hospital Admissions for Trauma and Acute Care Surgery. . Am Surg.

[11] Biz C, Buffon L, Marin R, Petrova N (2016). Orthopaedic nursing challenges in poly-traumatised patient management: A critical analysis of an Orthopaedic and Trauma Unit. . Int J Orthop Trauma Nurs..

[12] Morelli I, Luceri F, Giorgino R, Accetta R, Perazzo P, Mangiavini L (2020). COVID-19: not a contraindication for surgery in patients with proximal femur fragility fractures. . J Orthop Surg Res.

[13] Søreide K, Hallet J, Matthews J, Schnitzbauer A, Line P, Lai P, et al. Immediate and long-term impact of the COVID-19 pandemic on delivery of surgical services. Br J Surg. 2020;107(10):1250–61. 10.1002/bjs.11670. 10.1002/bjs.11670 PMC7267363 32350857

[14] Yang C, Ma QY, Zheng YH, Yang YX (2020). [Transmission routes of 2019-novel coronavirus (2019-nCoV)]. Zhonghua Yu Fang. Yi Xue Za Zhi.

[15] Hu Z, Song C, Xu C, Jin G, Chen Y, Xu X (2020). Clinical characteristics of 24 asymptomatic infections with COVID-19 screened among close contacts in Nanjing. China. Sci China Life Sci.

[16] Huang L, Zhang X, Zhang X, Wei Z, Zhang L, Xu J (2020). Rapid asymptomatic transmission of COVID-19 during the incubation period demonstrating strong infectivity in a cluster of youngsters aged 16–23 years outside Wuhan and characteristics of young patients with COVID-19: A prospective contact-tracing study. . J Infect.

[17] Han C, Duan C, Zhang S, Spiegel B, Shi H, Wang W, et al. Digestive Symptoms in COVID-19 Patients With Mild Disease Severity: Clinical Presentation, Stool Viral RNA Testing, and Outcomes. Am J Gastroenterol. 2020;115(6):916–23. 10.14309/ajg.0000000000000664. 10.14309/ajg.0000000000000664 PMC7172493 32301761

[18] Li J, Long X, Zhang Q, Fang X, Fang F, Lv X, et al. Emerging Evidence for Neuropsycho-Consequences of COVID-19. Curr Neuropharmacol. 2021;19(1):92–6. 10.2174/1570159X18666200507085335. 10.2174/1570159X18666200507085335 PMC7903490 32379592

[19] Mi B, Chen L, Xiong Y, Xue H, Zhou W, Liu G (2020). Characteristics and Early Prognosis of COVID-19 Infection in Fracture Patients. . J Bone Joint Surg Am.

[20] Samsami M, Zebarjadi Bagherpour J, Nematihonar B, Tahmasbi H (2020). COVID-19 Pneumonia in Asymptomatic Trauma Patients; Report of 8 Cases. . Arch Acad Emerg Med.

[21] Cipollaro L, Giordano L, Padulo J, Oliva F, Maffulli N (2020). Musculoskeletal symptoms in SARS-CoV-2 (COVID-19) patients. . J Orthop Surg Res.

[22] Oestern HJ, Tscherne H. Pathophysiology and Classification of Soft Tissue Injuries Associated with Fractures. In: Tscherne H, Gotzen L. eds. Fractures with Soft Tissue Injuries. Springer, Berlin, Heidelberg. 1984:1–9. 10.1007/978-3-642-69499-8_1.

[23] Peng L, Yang W, Zhangg D, Zhuge C, Hong L. Epidemic analysis of COVID-19 in China by dynamical modeling. medRxiv. 2020;02(16):20023465. 10.1101/2020.02.16.20023465.

[24] Wang J, Lu F, Zhou M, Qi Z, Chen Z (2020). [Tracheal intubation in patients with severe and critical COVID-19: analysis of 18 cases]. Nan Fang Yi. Ke Da Xue Xue Bao.

[25] Cook TM, El-Boghdadly K, McGuire B, McNarry AF, Patel A, Higgs A (2020). Consensus guidelines for managing the airway in patients with COVID-19: Guidelines from the Difficult Airway Society, the Association of Anaesthetists the Intensive Care Society, the Faculty of Intensive Care Medicine and the Royal College of Anaesthetists. . Anaesthesia.

[26] Chen R, Zhang Y, Huang L, Cheng BH, Xia ZY, Meng QT (2020). Safety and efficacy of different anesthetic regimens for parturients with COVID-19 undergoing Cesarean delivery: a case series of 17 patients. Can J Anaesth.

[27] Kumar Jain V, Lal H, Kumar Patralekh M, Vaishya R (2020). Fracture management during COVID-19 pandemic: A systematic review. . J Clin Orthop Trauma.

